# First Complete Genome Sequence of *Cucurbit Aphid-Borne Yellows Virus* from Pumpkin in the United States

**DOI:** 10.1128/MRA.01448-18

**Published:** 2019-02-07

**Authors:** Vivek Khanal, Akhtar Ali

**Affiliations:** a Department of Biological Science, The University of Tulsa, Tulsa, Oklahoma, USA; KU Leuven

## Abstract

*Cucurbit aphid-borne yellows virus* (CABYV) was first described in France in 1992 and since then has been reported in various parts of the world, including the United States. Here, we present the first complete genome sequence of a CABYV isolate (BL4) that was collected from pumpkin during the 2017 growing season in Oklahoma.

## ANNOUNCEMENT

Cucurbits are economically important cash crops in the United States and are mostly grown in the southern states, including Texas and Florida, which are the two major watermelon-producing states (1). CABYV belongs to the genus *Polerovirus* in the family *Luteoviridae* (2) and was first reported in France in 1992 (3). The virus causes yellowing and thickening of the older leaves in cucurbit plants and is often mistakenly attributed as a nutrient deficiency. Although the major veins of younger leaves would remain green after the infection, plant yield may be reduced (3). The virus is transmitted primarily by Aphis gossypii Glover and Myzus persicae Sulzer, and the transmission could be circulative, persistent, and nonpropagative (4, 5). CABYV has been reported from cucurbit crops across different climatic regions of the world such as temperate, Mediterranean, and subtropical (6), and no mechanical transmission has been reported (7). The main constraint for the management of diseases caused by members of *Luteoviridae* is that no effective strategy exists to cure plants after virus infection (8).

It has been nearly two and half decades since the first report of CABYV in the United States (9); however, to our knowledge, no complete genome sequence of any CABYV isolate from the United States has been reported so far. In this work, we report the first complete genome sequence of a CABYV isolate collected from a grower’s field in Oklahoma.

Previously, we reported CABYV for the first time from commercial cucurbit fields in Blaine County in Oklahoma (10). One of the dot-immunobinding assay (DIBA)-positive samples (10) against the CABYV antibody (designated as CABYV isolate BL4) was used in this work.

Total RNA was extracted from the CABYV-infected leaf tissues of pumpkin (11). Seven pairs of overlapping primers were designed (Table 1) and synthesized commercially (IDT Technologies, USA) from the previous CABYV isolates available from GenBank. All seven genome fragments were amplified by reverse transcription-PCR (RT-PCR) with the respective primer pairs using total RNA as the template, as described previously (11). Both 5′ end and 3′ end rapid amplification of cDNA ends (RACE) was performed using a commercial kit (TaKaRa Bio, Inc., Japan). Expected PCR products were analyzed and confirmed on 1% agarose gels and cleaned with Exosap-IT (Affymetrix). Purified PCR products were directly sequenced in both directions using an Applied Biosystems 3130 instrument.

**TABLE 1:** Primers used in RT-PCR to amplify the complete genome of *Cucurbit aphid-borne yellow virus*

Primer name	Primer sequence	Product size (nucleotides)
CABYVF1	ACAAAAGATACGAGCGGGTGA	665
CABYVR1	GCAAGTCGCCAAAAATCCAA	
CABYV F2	AGAGAGTCTGCTCCACGTGA	1,099
CABYV R2	GAGACTGTGCGTCTCCACTT	
CABYV F3	CGACCGCCGAAACAACCG	1,168
CABYVR3	TGCTCATCCGTAGACAAGCC	
CABYVF4	AACAAGCGCGAGATAGCGTT	998
CABYVR4	CGCCTCCCTGCATTTTGATT	
CABYVCP5	ATGAATACGGCCGCGGCTAGAAATC	600
CABYVCP5	CTATTTCGGGTTCTGGACCTGGCA	
CABYVF6	AACGGATCTTCCTCGGTTGC	1,320
CABYVR6	CACTTTCTCGTCTCTTCGTC	
CABYV F7	CACAGCCCACCCGGACTATA	544
CABYVR7	ACACCGAAACGCCAGGGG	

Nucleotide sequences of each genome fragment were confirmed and assembled using DNASTAR Lasergene 13 (Madison, WI) software. Sequences of all fragments were aligned using both Clustal W v2.1 (12) and Muscle v3.8.31 (13) alignment tools by joining overlapping sequences to generate the complete genome sequence of CABYV.

After the alignment, the complete genome sequence of the CABYV BL4 isolate is 5,679 nucleotides long (G+C content, 49.8%). Nucleotide BLAST searches showed that the CABYV BL4 isolate shares 85% to 97% nucleotide and 81% to 96% amino acid sequence identities with the available CABYV complete genomes in the GenBank database. The highest nucleotide (97%) and amino acid (96%) sequence similarities were observed with a Chinese isolate (GenBank accession no. EU000535) (14).

### Data availability.

The complete genome sequence of the CABYV BL4 isolate was deposited in GenBank under accession no. MK055337.
